# Neurotransmitter Modulation of Carotid Body Germinal Niche

**DOI:** 10.3390/ijms21218231

**Published:** 2020-11-03

**Authors:** Verónica Sobrino, Aida Platero-Luengo, Valentina Annese, Elena Navarro-Guerrero, Patricia González-Rodríguez, José López-Barneo, Ricardo Pardal

**Affiliations:** 1 Instituto de Biomedicina de Sevilla (IBiS), Hospital Universitario Virgen del Rocío/CSIC/Universidad de Sevilla, 41013 Seville, Spain; veronica.sobrino@imibic.org (V.S.); aplatero@us.es (A.P.-L.); vannese@us.es (V.A.); elena.navarroguerrero@ndm.ox.ac.uk (E.N.-G.); p-gonzalezrodriguez@northwestern.edu (P.G.-R.); 2 Departamento de Fisiología Médica y Biofísica, Universidad de Sevilla, 41013 Seville, Spain; 3 Centro de Investigación Biomédica en Red sobre Enfermedades Neurodegenerativas (CIBERNED), Spain

**Keywords:** carotid body, germinal niche, neurogenesis, angiogenesis, neurotransmitters, glomus cells, hypoxia, neuroblasts, mesenchymal progenitors, proliferation, differentiation, maturation

## Abstract

The carotid body (CB), a neural-crest-derived organ and the main arterial chemoreceptor in mammals, is composed of clusters of cells called glomeruli. Each glomerulus contains neuron-like, O_2_-sensing glomus cells, which are innervated by sensory fibers of the petrosal ganglion and are located in close contact with a dense network of fenestrated capillaries. In response to hypoxia, glomus cells release transmitters to activate afferent fibers impinging on the respiratory and autonomic centers to induce hyperventilation and sympathetic activation. Glomus cells are embraced by interdigitating processes of sustentacular, glia-like, type II cells. The CB has an extraordinary structural plasticity, unusual for a neural tissue, as it can grow several folds its size in subjects exposed to sustained hypoxia (as for example in high altitude dwellers or in patients with cardiopulmonary diseases). CB growth in hypoxia is mainly due to the generation of new glomeruli and blood vessels. In recent years it has been shown that the adult CB contains a collection of quiescent multipotent stem cells, as well as immature progenitors committed to the neurogenic or the angiogenic lineages. Herein, we review the main properties of the different cell types in the CB germinal niche. We also summarize experimental data suggesting that O_2_-sensitive glomus cells are the master regulators of CB plasticity. Upon exposure to hypoxia, neurotransmitters and neuromodulators released by glomus cells act as paracrine signals that induce proliferation and differentiation of multipotent stem cells and progenitors, thus causing CB hypertrophy and an increased sensory output. Pharmacological modulation of glomus cell activity might constitute a useful clinical tool to fight pathologies associated with exaggerated sympathetic outflow due to CB overactivation.

## 1. Introduction

The carotid body (CB), a paired organ located in the bifurcation of the carotid artery, is the main and prototypical polymodal arterial chemoreceptor in mammals. Despite its small size, the CB has relevant physiological functions due to its ability to detect changes in several physical and chemical parameters in the blood, such as pH, CO_2_ tension, glucose, lactate, temperature, and blood flow among others, to produce compensatory adaptive responses (see for a recent review [1]). An additional stimulus for CB activation of particular physiological relevance is the decrease in O_2_ tension in arterial blood (hypoxemia), a frequent condition faced by people who live at or travel to high altitudes and are thus exposed to low atmospheric air pressure and decreased O_2_ diffusion into the blood. CB activation is also critical to aid survival in patients with severe respiratory syndromes that cause a reduction of the O_2_ exchange capacity between the air and the pulmonary capillaries. A decline in arterial O_2_ tension is detected by CB O_2_-sensing cells, which rapidly (in few seconds) activate sensory fibers of the glossopharyngeal nerve impinging on neurons in the brainstem and autonomic centers to induce hyperventilation and increased heart rate. In this way, both O_2_ uptake and its distribution to the tissues are enhanced. Although there are other organs in the body acutely responding to hypoxia [2], activation of the CB is essential for organismal homeostasis because neurons in the respiratory center are insensitive to systemic changes in O_2_ tension. Indeed, bilateral removal of the CB in humans leaves individuals unaware of hypoxemia and with complete abolition of the hypoxic ventilatory response [3].

The CB parenchyma is organized into clusters of cells called glomeruli. Each glomerulus is considered an independent chemo-sensitive unit composed by four to eight neuron-like glomus cells, also called type I cells, which are richly innervated by sensory fibers of the petrosal ganglion and in close contact with a profuse network of fenestrated capillaries. Glomus cells are the O_2_-sensing elements in the CB; they contain abundant synaptic vesicles [4,5] with several neurotransmitters and neuropeptides (see below), which are rapidly released in response to hypoxia to activate the afferent sensory fibers. Glomus cells contain a broad variety of voltage- and ligand-gated ion channels, are electrically excitable, and can generate action potentials repetitively [6,7]. Hypoxia leads to glomus cell depolarization, extracellular Ca^2+^ influx and transmitter release due to modulation of O_2_-sensitive membrane ion channels [7,8,9,10,11]. Responsiveness to hypoxia is an intrinsic property of glomus cells, although it can be modulated by auto- and paracrine interactions within the glomerulus and by circulating agents such as cytokines or hormones (see [1]). The mechanisms underlying O_2_ sensing have remained elusive [12], although recent data strongly suggest that molecular specializations in glomus cell mitochondria confer upon them special sensitivity to hypoxia [13,14,15], with the generation of mitochondrial signals (NADH and reactive oxygen species) that modulate membrane ion channels [16,17]. In addition to the chemo-sensitive glomus cells, which are highly dopaminergic and can be easily identified using antibodies against tyrosine hydroxylase (TH), the CB glomeruli also contain a smaller number of type II, or sustentacular, cells with interdigitating processes that envelop glomus cells. Type II cells can be stained with antibodies against glial fibrillary acidic protein (GFAP), an intermediate filament commonly expressed in glial cells. Although type II cells were originally described as supportive cells, with little functional relevance, this view has completely changed in recent years (see below). Type II cells contribute to potentiation of the glomus cell-afferent sensory fiber synapse [18]. Moreover, type II cells, or a subpopulation of them, are tissue-specific adult multipotent stem cells that support CB structural plasticity [19]. In addition to the two main cell types, the CB contains intermediate progenitors, abundant macrophages, and fat cells.

## 2. The Carotid Body Germinal Niche

**Figure 1:**
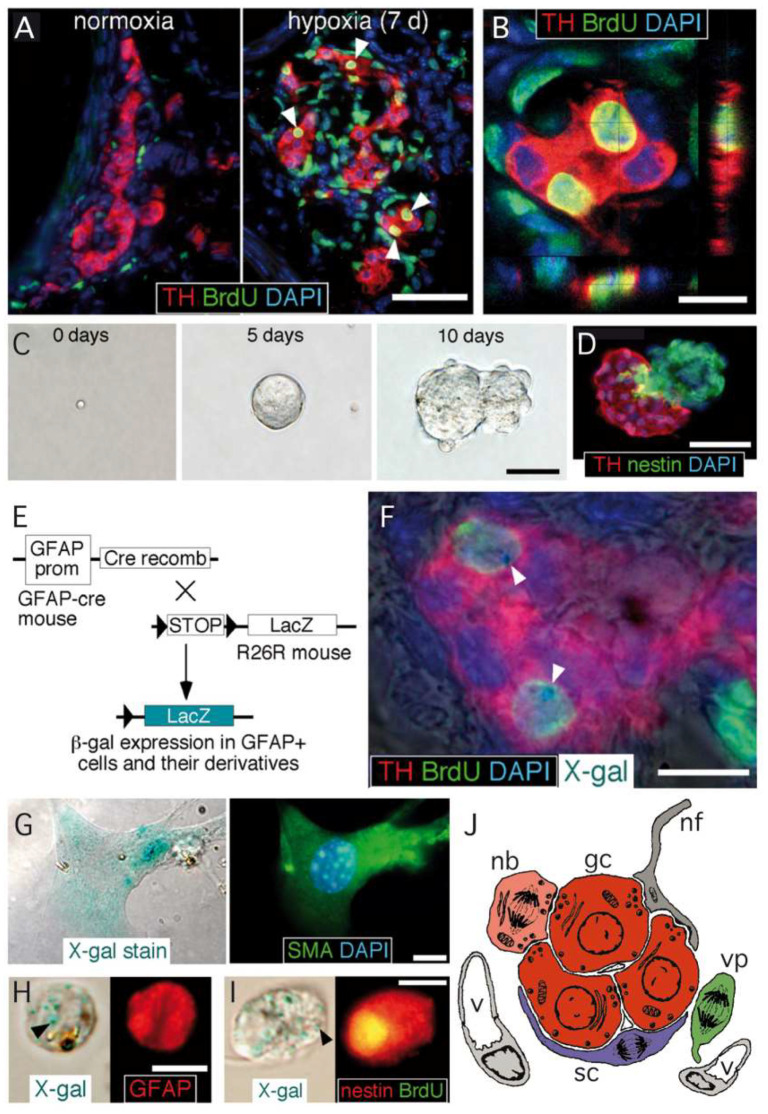
Carotid body germinal niche. (**A**) Immunocytochemical analysis of carotid bodies (CBs) removed from control mice (left) or from mice exposed to chronic hypoxia (10% O_2_) (right). The panels show the typical growth of the tyrosine hydroxylase (TH)-positive glomus cell mass after 7 days of hypoxia, the incorporation of BrdU to proliferating cells, and subsequent labeling of their differentiated progeny (white arrowheads). Scale bar, 50 µm. (**B**) Confocal microscope image illustrating the colocalization of BrdU-positive nuclei surrounded of TH-positive cytoplasm. Scale bar, 10 µm. (**C**) Sequential photographs of the same colony illustrating the formation of a typical neurosphere from a single CB stem cell. Scale bar, 50 µm. (**D**) Immunohistochemical analysis of a neurosphere thin section, illustrating the presence of nestin-positive progenitors within the neurosphere core and TH-positive glomus cells within a bleb budding out the neurosphere. Scale bar, 50 µm. (**E**) Mouse model used to investigate the glial lineage of the CB progenitors giving rise to glomus cells. The carotid bodies of GFAP-cre/floxed LacZ transgenic mice were analyzed by immunohistochemistry to look for type II cell derivatives, which appear as LacZ-positive. (**F**) Merge image of an immunohistochemical detection of TH, BrdU, and DAPI, with X-gal staining indicating. The presence of blue precipitates (arrowheads), illustrates that the two newly formed BrdU-positive glomus cells within this glomerulus are derived from GFAP-positive type II cells. Scale bar, 10 µm. (**G**) Magnified images illustrating the colocalization of X-gal deposits on smooth muscle actin (SMA)-positive smooth muscle cells. Scale bar, 5 µm. (**H**,**I**) Examples of X-gal-positive (arrowheads) cells dispersed from the CB of GFAP-cre/floxed LacZ mice during the initial 6 days of exposure to hypoxia. Cells are X-gal-positive and GFAP-positive (in **H**) or X-gal-positive, BrdU-positive, and nestin-positive (in **I**). Scale bar, 10 µm. (**J**) Diagram representing the most relevant cell types present in an adult carotid body glomerulus. nb: neuroblast; gc: glomus cell; sc: sustentacular cell; vp: vascular progenitor; nf: nerve fiber; v: vessel. Panels A–I modified from [19] and panel J modified from [28].

In parallel to its role in the acute detection of changes in blood O_2_ levels, the CB is essential for organismal adaptation to maintained hypoxemia, such as seen in high altitude residents [20,21,22], or in patients suffering chronic cardiopulmonary diseases [23,24]. In response to chronic hypoxia, the CB parenchyma grows several-fold its size, with a marked increase in the number of glomeruli, as well as a profuse angiogenesis [22,25] that facilitates irrigation of the newly generated chemoreceptor elements (Figure 1A,B). This process, which implies “neurogenesis” or production of new neuronal chemoreceptor cells, tunes the sensitivity of the organ to hypoxia, facilitating physiological reflex responses, such as the hypoxic ventilatory acclimatization [26,27].

**Figure 2:**
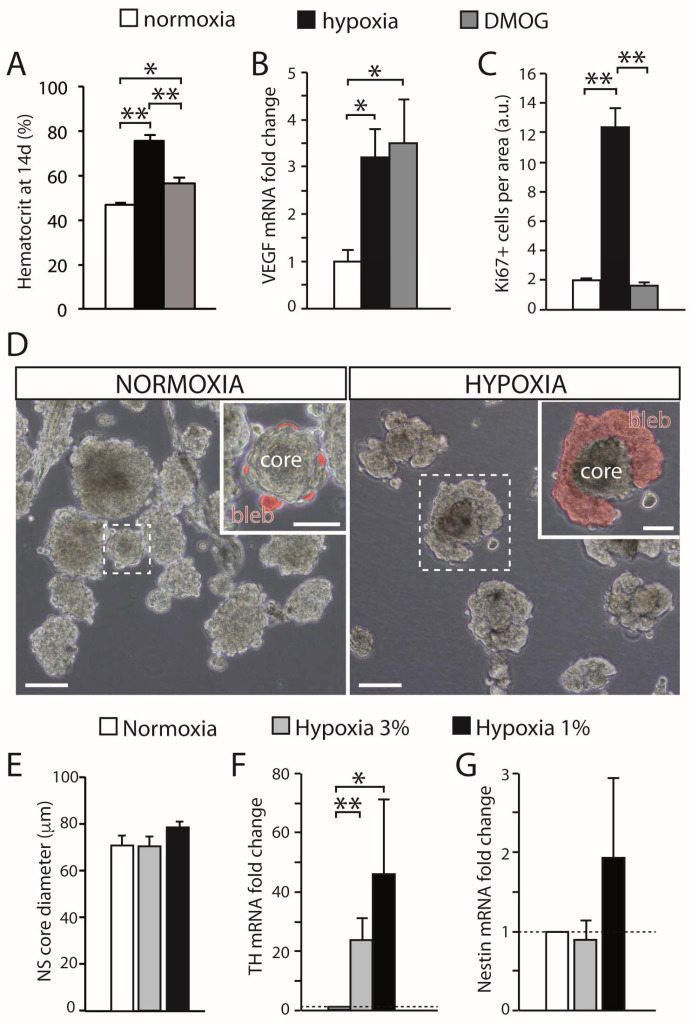
Effect of hypoxia on carotid body growth. (**A**) Quantification of the hematocrit in rats exposed to hypoxia (10% O_2_) or maintained in a normal atmosphere and treated with DMOG for 14 days, in comparison with normoxic rats. Note that inhibition of prolyl hydroxylases with DMOG increased the hematocrit, although to a lesser extent than hypoxia. (**B**) Quantitative PCR showing the induction of VEGF, a HIF-dependent gene, in the brain of rats exposed to normoxia, hypoxia, or treated with DMOG. (**C**) Quantification of the density of Ki67-positive cells within the CB parenchyma of rats exposed to normoxia (21% O_2_), hypoxia (10% O_2_), or DMOG (21% O_2_) for 3 days. DMOG did not increase cell proliferation in the CB despite the fact it induced HIF-dependent genes (3 rats per group). (**D**) Bright-field images of CB primary neurospheres cultured under normoxia (21% O2) or hypoxia (3% O2). Differentiating blebs of TH-positive cells are pseudo-colored in red in the insets. Scale bars: 100 µm (50 µm in insets). (**E**) Quantification of neurosphere core diameter in cultures grown at different levels of hypoxia (6 cultures for normoxia and hypoxia 3% O_2_, and 3 cultures for hypoxia 1% O_2_). (**F**,**G**) qPCR results showing relative expression of tyrosine hydroxylase (TH) and nestin in neurospheres at different levels of O_2_ tension. The expression of TH significantly increased under hypoxia, whereas nestin expression showed a non-significant variability (*n* = 7 cultures in normoxia and hypoxia 3% O_2_; 4 cultures for hypoxia 1% O_2_). Error bars are SEM. * *p* < 0.05; ** *p* < 0.01. Modified from [35].

CB growth in response to chronic sustained hypoxia is mainly due to activation of type II cells, which, as indicated above, are neural crest-derived, multipotent stem cells [19]. These cells contribute to both neurogenesis and angiogenesis by proliferating and differentiating into glomus and vascular cells, respectively [19,29,30]. CB stem cells (CBSCs) form clonal colonies (neurospheres) in culture (Figure 1C), where they recapitulate the formation of glomeruli with a core of nestin-positive progenitors and blebs of TH-positive cells (Figure 1D). These in vitro newly generated TH-positive cells resemble glomus cells in situ, as they express the characteristic voltage-gated ion channels and release neurotransmitters in response to hypoxia [19]. Cell fate-mapping experiments using transgenic mice (Figure 1E) strongly suggest that CB type II cells can differentiate into glomus (Figure 1F) and vascular cells, such as smooth muscle cells (Figure 1G). CB sustentacular cells achieve their germinal role by interchanging quiescent (GFAP-positive; Figure 1H) and proliferative (nestin-positive; Figure 1I) states, depending on the activity of the niche. Interestingly, although the structure of CB-derived neurospheres is similar in various species studied [31], the number of TH-positive cells in the blebs is much smaller in mice or humans than in the rat, suggesting that differentiation of type II cells into a glomus cell lineage may change according to animal species or environmental factors (see below). In addition to mature glomus cells and type II cells, the resting CB parenchyma also contains restricted progenitors belonging to either neuronal or vascular lineages. We have characterized a population of immature (TH-positive) neuronal cells or neuroblasts (nb in Figure 1J), present in the periphery of glomeruli, that have the capacity to proliferate and rapidly mature into new chemosensory glomus cells in response to hypoxia [32]. Similarly, we have also identified the presence of restricted vascular progenitors (vp in Figure 1J) that are resting in normoxia but rapidly proliferate and migrate towards blood vessels to contribute to angiogenesis in response to hypoxia [29,30,33]. A common anatomical feature shared by all these progenitor cells is their localization in the proximity of glomus cells. This structural disposition supports the hypothesis that glomus cells function as master regulators that, by means of the release of neurotransmitters, can modulate the activity of the various progenitors in the niche. In fact, we have shown that in vivo stabilization of HIFs by systemic administration of dimethyloxaloylglycine (DMOG), an inhibitor of prolyl-hydroxylases (PHDs), does not induce a CB cell proliferation similar to hypoxia (Figure 2A−C). This finding suggests that direct stabilization of HIFs in the hypoxic CB niche is not sufficient to get a full proliferative response, but rather some indirect signaling from glomus cells might be necessary. In agreement with this idea, experiments performed with neurospheres in vitro have shown that CB multipotent progenitor proliferation is insensitive to hypoxia (Figure 2D,E), even though these progenitor cells are able to increase dopaminergic differentiation in response to the lack of oxygen (Figure 2D−G). It has been reported that hypoxia-induced proliferation of some lineages in the CB niche, such as the immature neuronal lineage, is impaired in HIF2α-deficient mice [32,34], and that responsiveness of glomus cells to hypoxia is also strongly inhibited in this same mouse model [14]. Therefore, the available data suggest that the proliferative response of CB cells under hypoxia depends on the activation of O_2_-sensitive glomus cells and the release of paracrine mediators which activate neighboring stem cells and progenitors (see below). However, this response may be complemented with a direct action of hypoxia on progenitors of the various CB cell lineages [32].

As mentioned above, glomus cells form chemical synapses with sensory nerve endings of petrosal neurons (“chemosensory synapse”). They behave as presynaptic-like neurosecretory elements, containing a wide variety of neurotransmitters and modulators (ATP, adenosine, dopamine, histamine, serotonin, acetylcholine, erythropoietin, substance P, GABA, endothelin-1, or angiotensin II, among others) stored in secretory vesicles [11,36]. It is well established that ATP acts as the main neurotransmitter in the chemosensory synapse, operating through postsynaptic ionotropic (P2X2/3) purinergic receptors present in nerve endings [37,38]. However, the precise role of the majority of neuromodulators present in glomus cells is not completely known. Some transmitters seem to fine-tune chemosensory afferent signals [39,40,41], while others have an auto-inhibitory effect limiting glomus cell activation [42]. Given the exquisite sensitivity of glomus cells to lowering O_2_ tension, it is logical to suggest that they must play a fundamental role in the adaptive growth of the whole organ in response to hypoxia and that substances (neurotransmitters and neuromodulators) released by glomus cells regulate the activity of stem cells and progenitors in the CB germinal niche.

## 3. Neurotransmitter Modulation of Carotid Body Progenitor Cells

### 3.1. Carotid Body Multipotent Progenitors. Activation by Oxygen-Sensitive Glomus Cells

**Figure 3:**
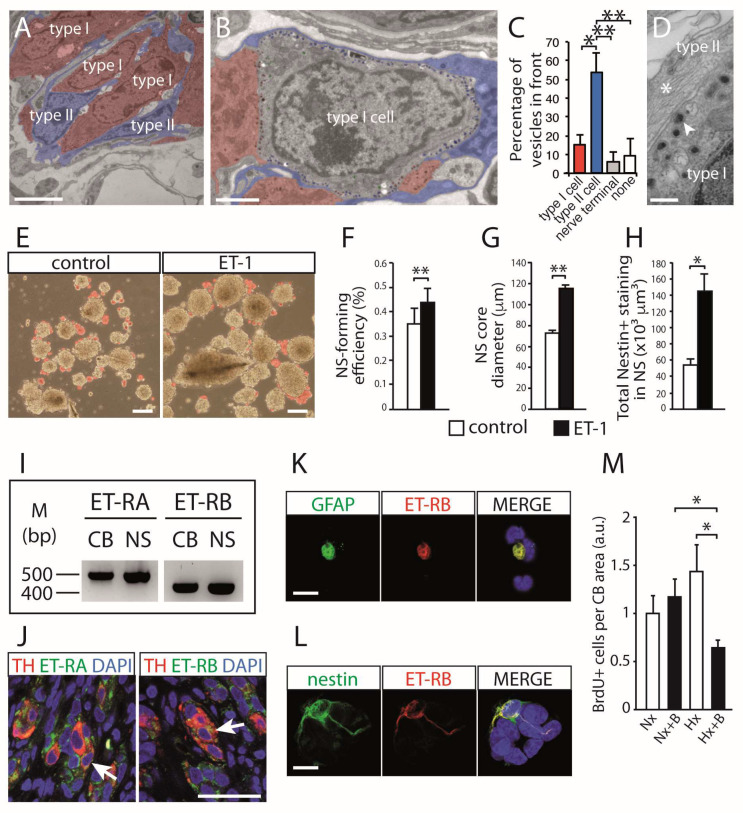
Carotid body multipotent stem cells and chemo-proliferative synapse. (**A**) Pseudocolored electron micrograph of a CB ultrathin section of a normoxic mice showing the close association of type II progenitor cells with glomus (type I) neuronal cells. Scale bar, 5 µm. (**B**) Pseudocolored electron micrograph illustrating the cellular elements surrounding a typical glomus cell within the CB parenchyma. Type II cells, and the type I vesicles facing them, appear in blue; type I cells, and the vesicles facing them, appear in red. Scale bar, 2 µm. (**C**) Quantification of the percentage of dense-core vesicles facing each of the different cellular elements surrounding a typical type I cell. (**D**) Electron micrograph of a CB slice, showing details of the contact area between type II and type I cells. Example of exocytotic vesicle apposed near the membrane is indicated with arrowhead. A bundle of intermediate filaments, characteristic of type II cells, is indicated by an asterisk. Scale bar, 2 µm. (**E**) Bright-field images of CB primary neurospheres showing the increase in their core diameter after 10 days of culture in the presence of ET-1. Differentiating blebs are pseudocolored in red. Scale bar, 100 µm. (**F**,**G**) Quantification of neurosphere-forming efficiency and neurosphere diameter from CB cultures (*n* = 4) treated with ET-1. (**H**) Quantification of nestin-positive staining within neurosphere sections from CB cultures (*n* = 3) treated with ET-1. (**I**) Non-quantitative PCR revealing mRNA expression of endothelin receptors A and B in whole CB neurospheres. (**J**) Confocal microscopy photographs of CB sections showing immunofluorescence detection of TH and endothelin receptors. Endothelin receptors A and B were both expressed in glomus cells (arrows). Scale bar, 50 µm. (**K**,**L**) Immunofluorescent detection of endothelin receptor B in GFAP-positive or nestin-positive cells dispersed from CB. Scales bars, 10 µm. (**M**) Quantification of BrdU-positive cells within CB sections of mice exposed to normoxia (Nx) or hypoxia (Hx) and treated systemically with (+B) or without Bosentan (five CBs per condition). Error bars represent SEM. * *p* < 0.05 and ** *p* < 0.01. Modified from [35].

Anatomical studies describing the ultrastructural arrangement of type II cells, or CBSCs, embracing glomus cells have suggested a direct communication between the two cell types. Glomus cells contain multiple secretory vesicles placed in front of the stem cell membrane (Figure 3A−D). The close juxtaposition of both membranes, with a narrow cleft, resembles the structure of a chemical synapse (Figure 3D), in which substances released from glomus cells act on type II cells [35]. Among the several substances tested, we have found that endothelin-1 (ET-1) has a powerful action on the biology of CBSCs. ET-1, secreted by glomus cells in response to hypoxia [43], noticeably increases proliferation of CBSCs, as shown by neurosphere assays in culture (Figure 3E−H). Both mRNAs of type A and type B ET-1 receptors are expressed in the neurospheres and in the whole CB (Figure 3I). Immunohistochemical and cytochemical detection of these receptors allowed us to prove their expression in glomus cells, as well as in GFAP-positive and nestin-positive progenitor cells (Figure 3J−L). Finally, systemic administration of an ET-1 receptor blocker (Bosentan) to hypoxic rats resulted in a clear decrease in proliferation rate of CB cells (Figure 3M), demonstrating that ET-1 is crucial to trigger stem cell-dependent activation of CB growth. ET-1 mediates cell proliferation in several tissues [44], and is required for the correct specification and migration of neural crest progenitors [45,46]. Moreover, the ET-1 gene is a typical hypoxia-responsive gene [47], and possesses hypoxia regulatory elements on its promoter [48]. By using transgenic mice to specifically sort CB glomus cells, we have shown expression and induction of ET-1 mRNA by hypoxia [35]. Therefore, although endothelial cells also produce ET-1 under hypoxia, the close anatomical vicinity of glomus and type II cells suggest that they form an O_2_-sensitive “chemoproliferative synapse”, such that the swift detection of hypoxia by glomus cells induces ET-1 release which is essential for the activation of CBSCs.

ET-1 is not the only glomus cell-secreted factor that modulates type II cell activity. Type II cells are non-excitable and display only a small outward K^+^ current [49]. However, they are activated by ATP secreted from glomus cells acting through P2Y metabotropic receptors, which in turn provokes Ca^2+^ release from internal stores [50]. Acetylcholine, serotonin, and angiotensin II, all of them released by CB glomus cells, also increase cytosolic Ca^2+^ in type II cells, acting on metabotropic receptors [51,52]. Intriguingly, the activation of type II cells by ATP produces ATP release to the external medium through pannexin-1 channels, a type of gap junction hemichannel highly expressed in these cells [53,54]. Hence, CB type II cells seem to have a dual functional role regulated by transmitters released from glomus cells. ATP-induced ATP release from type II cells contribute to potentiate the “chemosensory synapse” [18], whereas by means of the “chemoproliferative synapse” type II cells are activated to proliferate and differentiate into several cells types sustaining CB growth.

### 3.2. Angiogenesis and Glomus Cell-Released Vascular Cytokines

**Figure 4:**
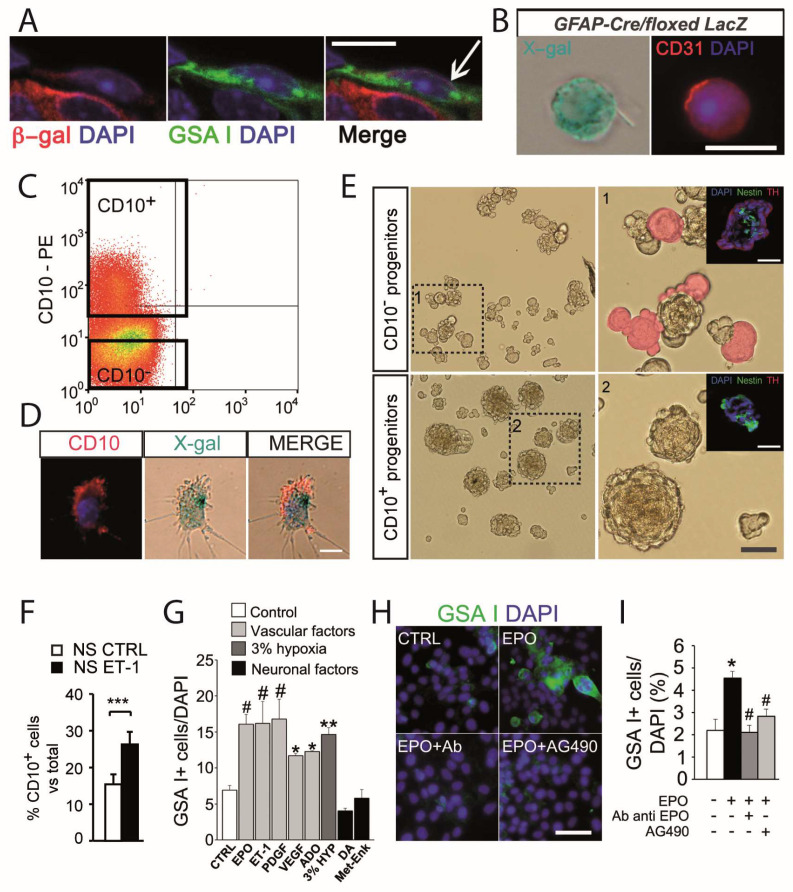
Carotid body progenitors of the mesenchymal lineage. (**A**) Confocal microscopy images from a CB section of a hypoxic GFAP-cre/floxed LacZ mouse, immunostained with an antibody against β-galactosidase (red) and stained with the endothelial marker GSA I (green). An endothelial cell (EC) derived from a GFAP-positive type II cell is pointed with an arrow. Scale bar, 10 µm. (**B**) Example of a GFAP-positive cell-derived CD31-positive EC, found in a CB cell dispersion from GFAP-cre/floxed LacZ mouse. Scale bar, 20 µm. (**C**) Dot-plot showing detection of CD10-positive cells in rat CB by flow cytometry. (**D**) Immunocytochemistry of dispersed CB cells from GFAP-cre/floxed LacZ transgenic mice to perform cell-fate mapping analyses to demonstrate that CD10-positive cells originate from CB multipotent cells. Scale bar, 10 µm. (**E**) Bright field pictures of neurospheres obtained in culture from CD10-positive FACS sorted cells. 1 and 2 show details of areas squared, with differentiated blebs pseudocolored in red. Insets in 1 and 2 show immunohistochemical images of CD10-negative (1) and CD10-positive (2) cell-derived neurosphere thin sections, stained for nestin (green) and TH (red). Scale bars, 50 µm. (**F**) FACS quantification of CD10-positive cells in dispersed neurospheres with and without ET-1 treatment. Error bars represent SEM *** *p* < 0.01. (**G**) Quantification graph indicating the average number of GSA-positive cells among total cells in different culture conditions. Error bars represent SEM. * *p* < 0.05 and ** *p* < 0.01; ^#^ *p* < 0.001, one-way ANOVA Newman–Keuls post hoc test compared to CTRL measurement. (*n* = 3 cultures). (**H**) Images displaying endothelial cell differentiation within adherent cultures of primary neurospheres in the presence of EPO, EPO combined with EPO neutralizing antibody, or EPOR inhibitor (AG490). Scale bar, 50 µm. (**I**) Quantification graph indicating the percentage of GSA I-positive cells in different culture conditions. Error bars represent SEM. * *p* < 0.05, one way ANOVA Newman–Keuls post hoc test compared to control measurement (white bar), and # *p* < 0.05, one way ANOVA Newman–Keuls post hoc test compared to EPO treated measurement (black bar) (*n* = 3 cultures). Panels A, B and G to H modified from [29], and panels C to F modified from [30].

Multipotent CBSCs not only give rise to new cells of neuronal lineage, but they also contribute to the strong angiogenic process that occurs in the CB during exposure to sustained hypoxia. CBSCs are able to differentiate into mesenchyme-like vascular cells, such as pericytes, smooth muscle, or even endothelial cells [29]. Based on the cell fate mapping approach previously used to study neurogenesis [19], we have demonstrated the contribution of sustentacular type II cells to the expansion of the CB endothelial cell compartment during hypoxia (Figure 4A,B) [29]. Our data suggest that one out of every three new endothelial cells formed under hypoxia is derived from CBSCs [29], indicating a relevant potential of CBSCs for endothelial differentiation. Interestingly, we have identified in the resting normoxic CB parenchyma a subpopulation of mesenchyme-restricted progenitors [30]. These cells express the cell surface marker CD10 (Figure 4C), are derived from GFAP-positive CBSCs (Figure 4D), and have lost the capacity to differentiate into the neuronal lineage in vitro (Figure 4E). Spheres obtained from these CD10-positive mesenchyme-restricted progenitors are larger than the ones obtained from multipotent cells, and display no signs of neuronal (TH-positive cell) differentiation (Figure 4(E1,E2)).

Proliferation of CB mesenchyme-restricted progenitors is sensitive to the presence of vascular cytokines. Treatment with ET-1 increases the proportion of CD10-positive cells within CB-derived neurospheres (Figure 4F) [30]. Moreover, differentiation of mesenchyme-restricted and multipotent progenitor cells into endothelial cells is clearly activated under the presence of ET-1, erythropoietin (EPO), adenosine (ADO), and others agents (Figure 4G). Among these signaling peptides, EPO seems to be particularly strong at activating mesenchymal differentiation (Figure 4H). The use of specific blocking antibodies, or intracellular signaling inhibitors, abrogates EPO-induced endothelial differentiation from CB progenitor cells (Figure 4H,I). Interestingly, we have shown that in addition to inducing proliferation, EPO is able to increase the migration capability of progenitor cells [33], suggesting an extra role when promoting angiogenesis. It is known that CB glomus cells produce important amounts of EPO, and its production is increased under a chronic hypoxic stimulus [55]. Hence, ET-1 and EPO are examples of vascular cytokines released by CB glomus cells that can activate proliferation of progenitors to direct their participation in angiogenesis.

### 3.3. Maturation of Carotid Body Neuroblasts in Response to Glomus Cell Activity

**Figure 5:**
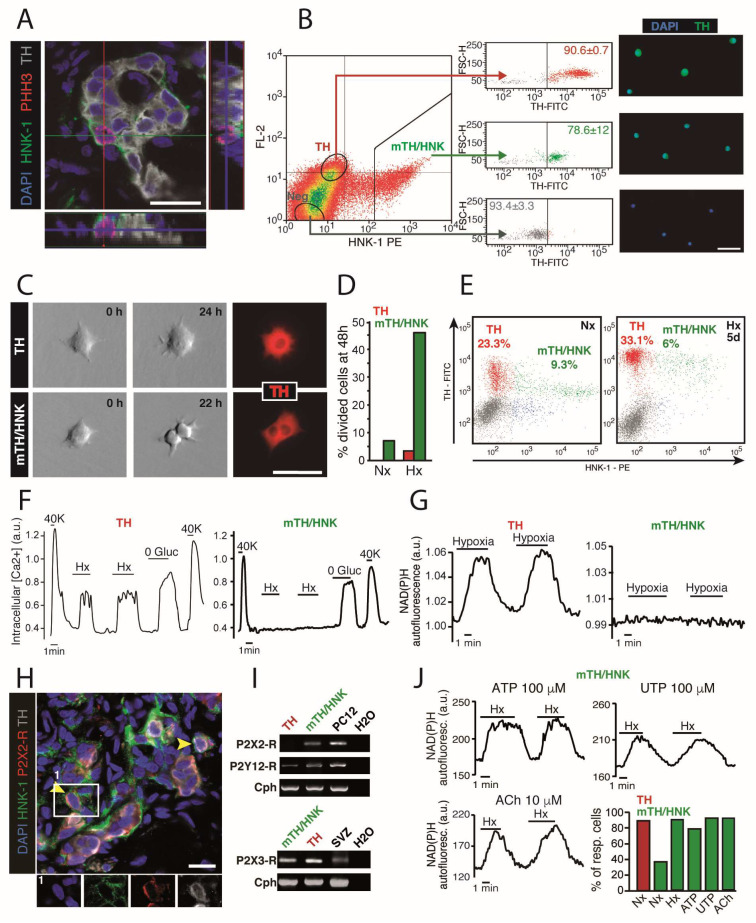
Carotid body neuroblasts. (**A**) Immunohistochemical analysis of CB from rats exposed to hypoxia for 48 h, illustrating the membrane expression of the glycoepitope HNK-1 in PHH3-positive proliferating CB neuronal (TH-positive) cells. Scale bar, 20 µm. (**B**) Left, FACS plot representing HNK-1 staining versus FL-2 channel autofluorescence, with sorting gates to enrich mature (TH; which are HNK-1-negative and autofluorescent) and immature (mTH/HNK; which are HNK-1-positive) CB glomus cells. Middle, TH expression in the different sorted cells, as revealed by intracellular staining of fixed cells, confirming the nature of different sorted populations. The percentages inside the plots refer to the proportion of cells within that particular side of the plot with regard to TH expression (*n* = 3 independent experiments with a total of 11 rats). Right, microscopy images of the different sorted cells, confirming their differential expression of the neuronal marker TH. Scale bar, 25 µm. (**C**) Time-lapse video microscopy imaging of TH-positive and mTH-positive/HNK-1-positive CB cells, sorted alive by flow cytometry (see **B**). After 48 h recording, most cell division activity was observed in the HNK-1-positive subpopulation. Scale bar, 25 µm. (**D**) Quantification of cell divisions observed after 48 h using time-lapse microscopy, confirming that proliferation is a feature of HNK-1-positive CB neuronal cells (*n* = 42 TH-positive and 42 mTH-positive/HNK-1-positive cells exposed to normoxia (Nx; *n* = 3 rats), and 96 TH-positive and 89 TH-positive/HNK-1-positive cells exposed to hypoxia (Hx; *n* = 5 rats)). Data in bar graph are represented as the sum of dividing cells among total cells studied. (**E**) Flow cytometry analysis of dispersed CB cells from normoxic (Nx) and 5 days hypoxic (Hx) rats, stained for TH and HNK-1. Note how mTH/HNK cells convert into TH (HNK-1-negative) mature glomus cells upon exposure to hypoxia. (**F**) Increases in intracellular calcium concentration in response to different stimuli, measured by changes in the fluorescence of the calcium indicator FURA-2. Note that unlike mature cells, CB neuroblasts (mTH/HNK) are insensitive to hypoxia although they respond to hypoglycemia (0 Gluc) or high potassium (40 K). (**G**) Monitoring of NAD(P)H autofluorescence generated by mitochondria in response to hypoxia in both mature glomus cells (TH) and neuroblasts (mTH/HNK). Note the absence of a response in neuroblasts. (**H**) Histological immunodetection of purinergic receptor P2X2 expression in CB neuroblasts (mTH-positive/HNK-1-positive). Scale bar, 25 µm. (**I**) RT-PCR detection of expression of different ionotropic and metabotropic purinergic receptors in both mature glomus cells (TH) and neuroblasts (mTH/HNK). (**J**) Increases in mitochondrial NAD(P)H in response to hypoxia in CB neuroblasts incubated with ATP (left up), UTP (right up) and Ach (left down), for 24-48 h, demonstrating maturation induced by purinergic and cholinergic signaling. The graph at the bottom right shows quantification of the percentage of cells responsive to hypoxia under the indicated conditions. Modified from [32].

Several groups have shown that proliferation of TH-positive cells greatly contributes to the growth of the glomus cell pool during the first few days in hypoxia [34,56,57,58]. However, we have shown that mature glomus cells in the rat, and probably also in other species, are post-mitotic and that the early glomus cell expansion seen in hypoxia is due to proliferation and maturation of a population of TH-positive neuroblasts, which differentiate into O_2_-sensitive glomus cells [32]. CB neuroblasts express human natural killer-1 (HNK-1), a typical neural crest cell surface marker (Figure 5A), and in resting conditions they are preferentially located in the periphery of CB glomeruli [32]. CB neuroblasts are smaller in size than mature glomus cells, have fewer secretory vesicles, and do not seem to establish synapses with the afferent nerve fibers [32]. The differential structural characteristics of TH-positive CB neuroblasts and mature glomus cells agree with the features of the two types of glomus cells within the CB parenchyma described in early literature [5,59,60]. We have set up a method to sort viable glomus cells and neuroblasts by flow cytometry (Figure 5B), so that we could study and compare their differential functional properties [32]. By using time-lapse microscopy, we confirmed that immature neuroblasts have the capacity to divide once or twice in response to the hypoxic stimulus (Figure 5C,D), whereas we barely observed any cell division in the sorted mature glomus cell population. When looking at the cellular composition of the CB of normoxic and hypoxic rats by flow cytometry (Figure 5E), we showed that HNK-1-positive and mild TH-positive neuroblasts (mTH/HNK; green dots in Figure 5E) move towards the TH-positive HNK-1-negative mature glomus cell population (TH; red dots in Figure 5E) in response to hypoxia [32]. Increasing expression of the TH enzyme might contribute to maturation of neuroblasts by increasing the amount of catecholamines available, and hence the number and functionality of secretory vesicles. Our analyses have also indicated that immature neuroblasts are O_2_-insensitive and do not exhibit the characteristic rise in intracellular Ca^2+^ (Figure 5F) [11], or increase in NAD(P)H autofluorescence (Figure 5G) [17], induced by hypoxia in glomus cells. These data suggest that the adult CB contains a subpopulation of immature glomus cells that in resting conditions do not contribute to sensory transduction, but in response to sustained hypoxia can rapidly proliferate and differentiate into mature O_2_-sensitive glomus cells to increase the sensory output of the organ [32].

Although proliferation and maturation of HNK-1-positive CB neuroblasts is accelerated by hypoxia in vitro, we have tested whether these processes are also influenced by paracrine signals from hypoxic glomus cells. Adenosine, serotonin, dopamine, or GABA, did not have a noticeable effect on neuroblast proliferation or maturation, although these agents are known to modulate the “chemosensory synapse” [41,42,61,62,63]. However, a potent and reproducible effect was seen with ATP and acetylcholine (ACh), which are also well-known transmitters stored in glomus cells. Flow cytometry-sorted CB neuroblasts acutely responded with increased cytosolic calcium concentration to the application of purinergic agonists (ATP or UTP) or ACh, and this response was abrogated by the addition of a purinergic receptor blocker [32]. Analysis of the expression of purinergic receptors in HNK-1-positive cells by immunohistochemistry and PCR (Figure 5H,I) demonstrated the presence of ionotropic (P2X2/3-R) and metabotropic (P2Y12) receptors in CB neuroblasts. Moreover, incubation of HNK-1-positive neuroblasts for 48 h in hypoxia, ATP, UTP (a potent agonist of purinergic receptors), or ACh, increased in all cases the number of immature cells that became acutely O_2_-responsive (Figure 5J). These data suggest that agents released from glomus cells facilitate proliferation and differentiation of neuroblasts towards a mature, O_2_-sensing cell phenotype.

## 4. Concluding Remarks

The CB, the main arterial O_2_ sensing organ, has an impressive structural plasticity, uncommon for an organ of the adult peripheral nervous system, due to the presence of a collection of multipotent stem cells as well as committed progenitors of different cell lineages. Glomus cells, the O_2_ sensing elements in the CB, play a central role in modulating the activity of the various cell types in the CB germinal niche to generate adaptive responses to hypoxia. However, maladaptive CB overactivation (and the resulting exaggerated sympathetic outflow) is known to participate in the pathogenesis of numerous diseases, such as hypertension, chronic heart failure, sleep apnea, or some forms of chronic kidney disease, which are frequent in the human population [64,65]. The list of sympathetic over-activation-related human pathologies in which a potential role of CB has been suggested can be extended to diabetes, obesity, metabolic syndrome, obstructive pulmonary disease, and asthma [12,65,66,67]. In most of these illnesses, an expansion in the size of the CB, very likely associated to disease progression, has been described [66]. Preclinical studies have shown that CB resection or deafferentation improves the symptoms [68,69,70]. However, translation of this procedure to the clinical setting has numerous hurdles and limitations [69]. An alternative therapeutic strategy is the development of pharmacological tools that abrogate CB growth without fully abolishing the chemoreceptive function of glomus cells. In this regard it would be interesting to explore the action of drugs that modulate differentially the “chemosensory” and “chemoproliferative” synapses, or even can separately inhibit activation of neuronal and vascular progenitors.
